# Ocular Complications after COVID-19 Vaccination, Vaccine Adverse Event Reporting System

**DOI:** 10.3390/vaccines10060941

**Published:** 2022-06-13

**Authors:** Cyril N. A. Nyankerh, Akosua K. Boateng, Mary Appah

**Affiliations:** 1 Department of Optometry and Vision Science, University of Alabama at Birmingham, Birmingham, AL 35294, USA; akoboat@uab.edu; 2 Department of Biostatistics, University of Alabama at Birmingham, Birmingham, AL 35294, USA; mappah5@uab.edu

**Keywords:** COVID-19, vaccination, ocular complications, adverse events, VAERS

## Abstract

In December 2020, the U.S. Food and Drug Administration licensed COVID-19 vaccines for emergency use authorization. We investigated the ocular adverse event reports in patients reported to the Vaccine Adverse Event Reporting System (VAERS) following vaccination against COVID-19. We searched the VAERS database for U.S. reports among persons who received COVID-19 vaccines between December 2020 and December 2021. Our goal was to analyze and quantify the ocular adverse events submitted to VAERS to provide clinicians and researchers with a broader view of these ocular side effects. During the analysis period, VAERS received 55,313 adverse event reports and, after data cleaning, 6688 reports met the inclusion criteria. Note that 2229 (33.33%) adverse events were classified as cases of eyelid swelling, ocular hyperemia and conjunctivitis, 1785 (26.69%) as blurred vision and 1322 (19.77%) as visual impairment. Females accounted for 73.8% of adverse event reports and the age group between 40 and 59 years had the most frequent adverse events. A higher proportion of these adverse events reported to VAERS was linked with the Janssen and Moderna COVID-19 vaccines. At the time of vaccination, a high proportion of patients reported conditions like allergies, hypertension, diabetes, thyroid disease, vascular and other autoimmune diseases. A review of these data suggests a possible association between COVID-19 vaccines and ocular adverse events. Physicians are cautioned not only to be aware of this potential problem, but to check any underlying patient conditions, and to carefully document in VAERS within a few weeks of vaccination. Future COVID-19 vaccine safety studies in healthy subjects would help clarify the vaccine’s safety profile.

## 1. Introduction

In December 2020, the first COVID-19 vaccine known as Pfizer-BioNTech (Pfizer) COVID-19 Vaccine was approved by the U.S. Food and Drug Administration (FDA) for emergency use authorization. Since then, other vaccines that use vectors (Ad26.COV2, Janssen Johnson & Johnson [1]; ChAdOx1 nCoV-19/AZD1222, Oxford-AstraZeneca [2]), mRNA (mRNA-1273, Moderna) [3] and protein subunits (NVXCoV2373, Novavax) [4] have also been approved for the prevention of COVID-19 disease. As of 23 December 2021, the United States had confirmed 51,574,787 cases of COVID-19 and 809,300 deaths. Despite the tendency for the vaccines to prevent people from getting severely ill from COVID-19, serious adverse events following vaccination have been widely reported and are carefully being monitored [4,5]. To date, little is known about potential ocular-specific complications post-vaccination. Even though vaccines are known to aid in developing immunity by eliciting immunological mechanisms, they also bear the risk of immune-mediated adverse effects on all parts of the human body. Since the number of vaccinations will continue to increase in the following months, eye care practitioners need to be aware of potential ocular adverse events of the vaccine. For example, some studies have reported cases of neuropathy with panuveitis [6], retina artery occlusions, venous stasis retinopathy and non-arteritic anterior ischemic optic neuropathy [7] following administration of the COVID-19 vaccine. Ocular adverse events are not unique to COVID-19 vaccines since history has shown that vaccines for hepatitis B, polio, yellow fever, H1N1 and influenza are associated with bilateral optic neuritis, uveitis, chorioretinitis, acute and idiopathic maculopathy [8,9,10,11,12]. Surprisingly, respective clinical trial results from the COVID-19 vaccines have suggested rare adverse effects on the eye. Considering the wide-scale campaign for COVID-19 vaccination globally, adverse ocular reactions remain a concern. Eye care providers must therefore be alert to potential post-COVID-19 vaccination adverse reactions. Awareness of post vaccination adverse events was key to the development of the VAERS by the U.S. Food and Drug Administration (FDA) in 1990 as a national early monitoring system for vaccine safety. While the literature has provided a partial epidemiological picture in the form of isolated case-study reports, none has attempted to provide a broader view of these ocular adverse events. We analyzed the VAERS registry to provide a broader view of these ocular adverse events following vaccination [13]. The goal of this study was to investigate the VAERS, quantify and describe ocular adverse events and symptoms after vaccination with the Pfizer-BioNTech, Moderna and Janssen COVID-19 vaccines. We were particularly interested in characterizing the adverse events among patients who were considered normal or had stable and well-controlled coexisting conditions at the time of vaccination. Our descriptive analysis of reports to VAERS of ocular complications after COVID-19 vaccines will provide physicians and researchers with data on common ocular adverse events to look out for in their vaccinated patients.

## 2. Materials and Methods

The FDA VAERS is an early warning system overseen by the U.S. Department of Health and Humans Services (DHHS) that can detect potential issues with licensed vaccines by recording and analyzing adverse events following vaccinations. These adverse event reports can be submitted by health care providers who administer vaccines and vaccine manufacturers licensed in the USA [14]. Raw VAERS is publicly available and can be directly assessed or downloaded through the CDC WONDER (Center for Disease Control and Prevention Wide-ranging Online Data for Epidemiologic Research) search tool (https://wonder.cdc.gov/vaers.html, accessed on 5 June 2022) [15]. All reported ocular adverse events following the Pfizer-BioNTech, Moderna and Janssen vaccines that had been processed as of 22 December 2021 were first queried using the CDC WONDER tool. The key terms “Eye”, “Ocular” or “Ophthalmic” were used to select all symptoms. Since each adverse event report can contain multiple symptoms related to the patient’s clinical presentation, the list of unique symptoms was reviewed and categorized by two optometrists, C.N. and A.B. For example, cases of gaze palsy, double vision and lazy eye were categorized under “Extraocular muscle paresis/ophthalmoplegia”. Differences in interpretations between the reviewers were internally adjudicated. Each diagnosis was then queried through the CDC WONDER tool to obtain specific details about the related adverse event report. All adverse events had the name of the vaccine manufacturer recorded in VAERS, so we included the variable “Vaccine manufacturer” in our analysis. On the other hand, we were unable to get adequate data associated with vaccine doses and the number of boosters administered, so we excluded the number of doses from our analysis. We included only patient data with ages above 18 years in our analyses. Our data were analyzed in relation to patient age, current illness, allergies/health history, type of vaccine administered, ocular signs, current illness, symptoms and diagnosis. Using MATLAB software, we searched the VAERS for the keyword ‘‘Normal’’ under the “Current illness” tab in “report narratives” to identify cases of patients who were normal or had stable and well-managed conditions at the time of vaccination. Any patient record that showed the presence of an ongoing and uncontrolled medical condition was excluded from the study. If a patient record had duplicate submissions, we deleted all but the most sight-threatening record per patient as examined by our team of clinicians. We also discarded any case record with missing or “unknown” entries for any of the variables studied. Reports submitted to a passive surveillance system like VAERS tend to underestimate the true incidence of an event and are often incomplete, inaccurate and/or otherwise biased [9]. Consequently, much of the information derived from such reports is properly restricted to descriptive summarization and formal statistical analyses only in selected circumstances where uncertainties concerning the data do not preclude them.

Ethical considerations: The institutional board of UAB approved this study and waived the need for informed consent for the use of these de-identified patient data. All adverse event data were publicly available from VAERS. The study adhered to the Declaration of Helsinki as well as all federal laws. This study also followed the Strengthening the Reporting of Observational Studies (STROBE) in Epidemiology reporting guideline [16].

## 3. Results

**Figure 1:**
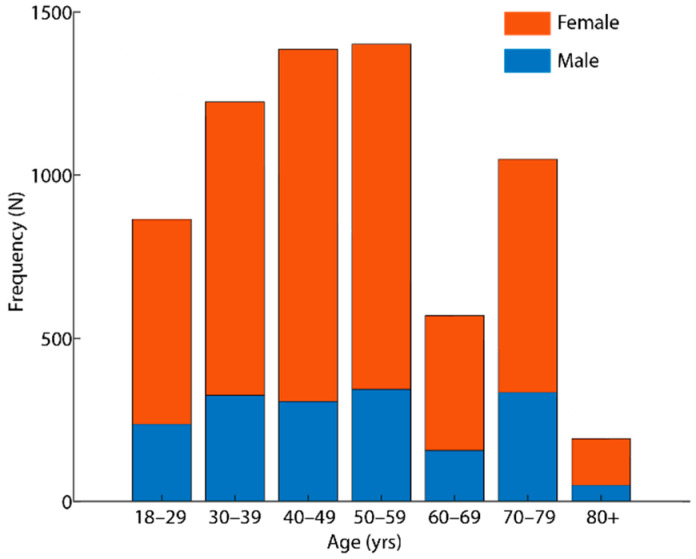
Frequency of ocular adverse events by age after vaccination against COVID-19 in 6688 reports to the VAERS database, 2020–2021.

The initial search of the VAERS database yielded 55,313 adverse event reports submitted to VAERS between December 2020 and December 2021. Of these, 6688 (4934 females and 1754 males) met the inclusion criteria. The ages ranged from 18 to above 80 years, with a mode of 50–59 years (Figure 1). Across all age groups, the number of ocular adverse events reported was higher in females. At the time of the study, the CDC reports showed that a total of 500, 222, and 330 vaccine doses had been administered, with 204.7 million people being fully vaccinated, out of which another 64.5 million people had received a booster dose.

The ocular adverse events reported to VAERS are shown in Table 1. The most-reported events were “Eye swelling, ocular hyperemia, conjunctivitis (33.33%)”, “blurred vision (26.69%)” and “visual impairment (19.77%)”. These conditions together accounted for more than 70% of all complications. More than 1% of the adverse events were related to eye hemorrhages (retina in nature) among patients with blood clot disorders. The proportion of females in the VAERS database with ocular adverse events was significantly higher for cases of Blepharospasm ($\chi_{1}^{2}$ = 6.67, *p* = 0.009), Eyelid swelling, ocular hyperemia, conjunctivitis ($\chi_{1}^{2}$ = 71.20, *p* < 0.0001) and Photophobia ($\chi_{1}^{2}$ = 4.57, *p* = 0.003) whereas males had higher ocular adverse events for Extraocular muscle paresis/ophthalmoplegia ($\chi_{1}^{2}$ = 97.27, *p* < 0.001), Eye contusion ($\chi_{1}^{2}$ = 6.69, *p* < 0.05) and Eyelid ptosis ($\chi_{1}^{2}$ = 8.36, *p* < 0.005).

To determine the vaccine types associated with these ocular complications, we grouped the different adverse events according to vaccine manufacturer/name (Table 2). Out of the patients who had ocular complications, 3346 (50%) received the Pfizer-BioNTech vaccine, 2552 (38.2%) received the Moderna vaccine and 790 (11.8%) received the Janssen vaccine. The ocular adverse events were significantly associated with the administered vaccines ($\chi_{26}^{2}$ = 135.10, *p* < 0.0001). From Table 2, we observed that the ocular adverse events (“Blurred vision”, “Eyelid swelling, ocular hyperemia conjunctivitis”, and “Visual Impairment”) that had relatively high numbers associated with the Janssen vaccine (36.33%, 19.5%, 23.04%) also had high numbers associated with the Pfizer (25.94%, 34.91%, 19.07%) and Moderna (24.69%, 35.54%, 19.67%) vaccines, respectively. The proportion of the vaccine type administered was not equal for Blurred vision ($\chi_{2}^{2}$ = 43.71, *p* < 0.0001), Extraocular muscle paresis/ophthalmoplegia ($\chi_{2}^{2}$ = 10.81, *p* = 0.004), Eye hemorrhage ($\chi_{2}^{2}$ = 7.62, *p* = 0.02), Eyelid swelling, ocular hyperemia, conjunctivitis $(\chi_{2}^{2}$ = 77.42, *p* < 0.0001) and Visual Impairment $(\chi_{2}^{2}$ = 7.08, *p* = 0.029). Among the aforementioned, the Janssen vaccine had a significantly higher proportion of adverse events for all cases except for Eyelid swelling, ocular hyperemia, conjunctivitis, which had a higher proportion of adverse events associated with the Moderna vaccine. Since patients whose conditions were being managed without significant medical issues were considered normal, we sought to find out the controlled conditions and medications that were highly reported. From the word cloud that was created, the leading conditions were allergies, hypertension, diabetes, thyroid disease, vascular and other autoimmune diseases like rheumatoid arthritis. Also, the most common medications that were taken by patients at the time of vaccination were multivitamins, levothyroxine, blood pressure medications (Lisinopril, Amlodipine, Losartan), Metformin, Antibiotics, and allergy medications (data not shown).

## 4. Discussion

Though some clinicians and researchers have reported adverse ocular effects of COVID-19 vaccinations, all of them have been in the form of isolated case reports [17,18,19]. The primary goal of this study was to provide a broader picture and evaluate the presence of ocular adverse events following COVID-19 vaccination. To the best of our knowledge, this is the first study to address this issue using the nationwide database, VAERS. Even though ocular complications following vaccination are relatively rare, we found 14 main ocular adverse events. These ocular events have also been reported in patients who have suffered the COVID-19 disease [20], which may suggest a shared common pathway of the ocular complications associated with the COVID-19 disease and post-vaccination events. The most reported adverse event was conjunctiva-related, which together accounted for 33.33% of all cases. Similar to our findings, a literature review of all ocular adverse case reports published from vaccines against other harmful diseases showed that conjunctiva and eyelid reactions are the most reported in post-marketing registries [21]. From the data presented in Table 2, it appears that all the different vaccine types administered in the USA were associated with ocular adverse events. These data can be easily interpreted as Pfizer vaccines were more associated with the ocular adverse events, but that cannot be established since the Pfizer vaccine was the most administered in our dataset and, hence, only a test of proportion could establish that premise. To test the null hypothesis that the vaccines administered were associated with ocular adverse events, we performed a chi-square test of association. Surprisingly, the Janssen and Moderna vaccines were mostly associated with the reported ocular adverse events in VAERS. There had been prior reports of vascular-related events after receiving the Janssen vaccine by the CDC COVID-19 response team [22]. This finding is similar to the higher proportion of vascular events in our study that was seen as hemorrhages in the eye associated with the Janssen vaccine. However, emphasis must be made that, since we did not have data showing the number of doses or boosters per vaccine type administered to each patient, trying to establish a cause–effect relationship will be implausible. Also, since VAERS does not include an unvaccinated group, we could not calculate rates nor determine if the vaccines themselves are associated with an increased risk of adverse events. The less-reported but sight-threatening ocular events included eye hemorrhages which were of retina origin (1.42%), ophthalmic herpes zoster/simplex (0.64%) and extra ocular muscle paresis (4.98%). Likewise, these events have been reported in COVID-19 patients too [23,24,25]. Surprisingly, cases of ocular contusion were also reported (0.85%) but, upon further investigation, we noticed that a significant proportion of these patients had symptoms of lightheadedness, headaches, dizziness, and numbness, which could have led to hitting their eyes against an obstacle. After stratifying all cases by age group, we identified a possible association between COVID-19 vaccines and the incidence of ocular events. The age groups that reported the most effects were those between 40 and 59 years. It is interesting to note that symptoms of tachycardia were common among females (42/44). The most common cause of drug allergies among the vaccinated patients reported to VAERS were antibiotics like Bactrim, Amoxicillin, Penicillin, and sulfa-containing drugs. Immune-mediated adverse event reactions to vaccines have been found in patients who are also allergic to these antimicrobials [26]. The limitations of our study include those common to any passive reporting system including underreporting [27], lack of denominator of doses administered to calculate rates, biased and stimulated reporting [28], which hinders our ability to establish causality. Additionally, since anyone can submit information to VAERS, there is a potential that the quality of information in terms of amount and accuracy might be affected. The present study monitored short-term post-vaccination side effects; therefore, real-world studies for longer periods need to be carried out among a larger pool of participants to assess the long-term side effects and the post-marketing surveillance of COVID-19 vaccines. Having noted this, we suggest that reports of these adverse events should continuously be verified against other ancillary testing and clinical examinations if a causal relationship is to be asserted. 

## 5. Conclusions

Overall, the benefits of COVID-19 vaccination both at an individual and a population level far outweigh the risks of ocular complications. Eye care professionals and other clinicians should be aware of these rare but possible ocular adverse effects after COVID-19 vaccination, especially those that are sight-threatening in nature. Also, patients who have a history of allergic/autoimmune reactions against medications like antimicrobials should be carefully and closely monitored post vaccination. Prospective research will be needed to establish any causal relationship between COVID-19 mRNA, viral vector vaccines and ocular complications, particularly as new strains of the virus emerge, and new vaccines are being developed as booster shots to combat them. Future vaccine safety studies by vaccine manufacturers should consider the ocular adverse events in clarifying the vaccine’s safety profile. 

## Figures and Tables

**Table 1:** Proportion of main adverse events.

Adverse Event	Count (N)	Percentage
Blepharospasm	145	2.17
Blurred vision	1785	26.69
Dry eye	64	0.96
Exophthalmos	18	0.27
Extraocular muscle paresis/ophthalmoplegia	333	4.98
Eye contusion	57	0.85
Eye hemorrhage	95	1.42
Eye hypoaesthesia	48	0.72
Eyelid swelling, ocular hyperemia, conjunctivitis	2229	33.33
Eyelid ptosis	102	1.53
Ophthalmic herpes zoster/simplex	43	0.64
Photophobia	265	3.96
Visual impairment	1322	19.77
Vitreous floaters	182	2.72
Total	6688	100.00

**Table 2:** Main adverse events grouped by the type of vaccine administered.

Diagnosis	Vaccine Type	Group Count
Blepharospasm	Janssen	21
	Moderna	51
	Pfizer	73
Blurred vision	Janssen	287
	Moderna	630
	Pfizer	868
Dry eye	Janssen	2
	Moderna	22
	Pfizer	40
Exophthalmos	Janssen	5
	Moderna	6
	Pfizer	7
Extraocular muscle paresis/ophthalmoplegia	Janssen	57
	Moderna	129
	Pfizer	147
Eye contusion	Janssen	7
	Moderna	23
	Pfizer	27
Eye hemorrhage	Janssen	18
	Moderna	41
	Pfizer	36
Eye hypoesthesia	Janssen	1
	Moderna	18
	Pfizer	29
Eyelid swelling, ocular hyperemia, conjunctivitis	Janssen	154
	Moderna	907
	Pfizer	1168
Eyelid ptosis	Janssen	4
	Moderna	38
	Pfizer	60
Ophthalmic herpes zoster/simplex	Janssen	2
	Moderna	18
	Pfizer	23
Photophobia	Janssen	31
	Moderna	105
	Pfizer	129
Visual impairment	Janssen	182
	Moderna	502
	Pfizer	638
Vitreous floaters	Janssen	19
	Moderna	62
	Pfizer	101

## Data Availability

All data on reports of ocular adverse events submitted to the U.S. Food and Drug Administration’s Vaccine Adverse Events Reporting System are available for download from: https://wonder.cdc.gov/vaers.html (accessed on 22 December 2021).
